# Clinical outcome of conservative versus surgical management in acute intestinal obstruction

**DOI:** 10.6026/973206300215025

**Published:** 2025-12-15

**Authors:** Sandeep Sharma, Kaushlendra Singh Narwariya, Rakesh Shakya, Upendra Singh, Vishnu Kumar Gupta

**Affiliations:** 1 Department of General Surgery, SRVS Medical College Shivpuri, Madhya Pradesh, India; 2 Department of General Surgery, Government Medical College, Satna, Pradesh, India; 3 Department of Community Medicine, SRVS Medical College Shivpuri, Madhya Pradesh, India

**Keywords:** Acute intestinal obstruction, conservative management, surgical management, clinical outcome, hospital stay

## Abstract

Acute intestinal obstruction presents a clinical challenge, and the optimal management approach remains uncertain. Therefore, it is of interest to compare outcomes of conservative versus surgical management in 100 patients with acute intestinal obstruction. Conservative treatment successfully resolved most cases (86.5%) and resulted in significantly shorter hospital stay. Surgical management was required for complicated or non-responsive cases and showed a higher rate of postoperative complications. Mortality remained low and comparable between both groups. Thus, we show that conservative management is effective for most patients with acute intestinal obstruction, resulting in shorter hospital stays, while surgery remains crucial for complicated or non-responsive cases, with comparable mortality rates between both treatments.

## Background:

Acute intestinal obstruction is a prevalent and potentially life-threatening condition that necessitates timely diagnosis and appropriate management. It is characterized by the interruption of normal bowel transit, leading to symptoms such as abdominal pain, vomiting, distension and constipation. The etiology of acute intestinal obstruction varies, with postoperative adhesions being the most common cause, followed by hernias, malignancies and inflammatory conditions [1]. The management of acute intestinal obstruction can be broadly categorized into conservative and surgical approaches. Conservative management typically includes bowel rest, nasogastric decompression, intravenous fluid resuscitation and correction of electrolyte imbalances. This approach is often effective in cases of partial obstruction without signs of strangulation or ischemia [2]. However, surgical intervention becomes imperative when there is evidence of bowel ischemia, perforation, or failure of conservative measures. Surgical options range from adhesiolysis to bowel resection, depending on the underlying cause and severity of the obstruction [3]. Recent studies have highlighted the importance of early and accurate diagnosis in determining the appropriate management strategy. Imaging modalities, particularly computed tomography (CT), play a crucial role in identifying the site and cause of obstruction, as well as assess for complications such as ischemia or perforation [4]. Despite advancements in diagnostic techniques and management protocols, the optimal approach for managing acute intestinal obstruction remains a subject of ongoing research. Factors influencing the decision between conservative and surgical management include the patient's clinical presentation, underlying comorbidities and the presence of complications [5]. Therefore, it is of interest to evaluate and compare the clinical outcomes of conservative versus surgical management in patients with acute intestinal obstruction, providing insights into the efficacy and safety of each approach in a contemporary clinical setting.

## Methodology:

This hospital-based, prospective observational study was conducted in a tertiary care teaching hospital. A total of 100 patients aged 18 years and above, diagnosed based on clinical features and radiological findings, were included. The diagnosis was established based on characteristic symptoms, including abdominal pain, distension, vomiting and absolute constipation, as well as radiological evidence of air-fluid levels or dilated bowel loops on plain abdominal radiographs, ultrasonography, or computed tomography of the abdomen. Patients with chronic or subacute intestinal obstruction, postoperative obstruction occurring within ten days of abdominal surgery, malignancy- associated obstruction, or perforation peritonitis were excluded. Informed written consent was obtained from all participants before inclusion. Of the total enrolled patients, 52 were managed conservatively and 48 required operative intervention, depending on their clinical response and disease severity. Conservative treatment consisted of nasogastric decompression, intravenous fluid resuscitation, correction of electrolyte imbalance and bowel rest with close monitoring of vital signs and abdominal findings. Serial examinations and repeat radiographs were performed to assess improvement. Patients who failed to show clinical or radiological resolution within forty- eight to seventy-two hours, or who developed signs of peritonitis or strangulation, were taken up for surgery. Operative procedures included adhesiolysis, resection and anastomosis, or release of volvulus, depending on intraoperative findings and bowel viability. All relevant data, including demographic details, clinical presentation, investigation results, type of management, intraoperative findings, complications, duration of hospitalization and final outcome, were recorded in a structured proforma. The primary outcome assessed was complete clinical recovery without recurrence of obstruction during the hospital stay, while secondary outcomes included postoperative complications, length of hospital stay, requirement for re-intervention and mortality. Data analysis was performed using the Statistical Package for the Social Sciences (SPSS) software version 26.0. Quantitative variables were expressed as mean ± standard deviation and categorical variables as frequency and percentage. The Chi-square test and Student's t-test were applied to assess statistical significance, with a p value less than 0.05 considered significant.

## Results:

A total of 100 patients with acute intestinal obstruction were included in the study, of whom 52 were managed conservatively and 48 underwent surgical intervention. The mean age of patients in the conservative group was 46.3 ± 14.2 years, while in the surgical group it was 48.7 ± 13.5 years, showing no statistically significant difference (p = 0.35). Males predominated in both groups, with 31 (59.6%) in the conservative group and 29 (60.4%) in the surgical group and the difference in sex distribution was not significant (p = 0.84). The mean body mass index (BMI) was comparable between the two groups (23.1 ± 3.2 kg/m^2^ vs. 23.8 ± 3.5 kg/m^2^, p = 0.28) (Table 1 - see PDF). The clinical presentation was similar across the groups. Abdominal pain was the most common symptom, reported in 97.0% of patients overall, followed by abdominal distension (90.0%), vomiting (80.0%) and constipation (90.0%). There were no statistically significant differences between the conservative and surgical groups for any presenting symptom (Table 2 - see PDF). Adhesions were the leading cause of obstruction, accounting for 50% of cases, followed by hernias (20%), volvulus (14%), tumors (8%) and other causes including intussusception (8%). Distribution of etiology was broadly similar between the two groups, with a slightly higher proportion of hernia and volvulus in the surgical group (Table 3 - see PDF). Among patients managed conservatively, 45 (86.5%) showed complete resolution without the need for surgical intervention. The mean hospital stay was significantly shorter in the conservative group (5.2 ± 1.8 days) compared to the surgical group (9.6 ± 3.1 days, p <0.001). Postoperative complications occurred in 12 (25.0%) patients in the surgical group, while 7 (13.5%) patients in the conservative group required subsequent surgical intervention due to failure of conservative management. Mortality was low in both groups, with one death (1.9%) in the conservative group and two deaths (4.2%) in the surgical group, with no statistically significant difference (p = 0.57) (Table 4 - see PDF).

## Discussion:

This study aimed to evaluate and compare the clinical outcomes of conservative versus surgical management in patients with acute intestinal obstruction. Our findings suggest that conservative management is effective in a significant proportion of patients, leading to shorter hospital stays and favorable clinical outcomes. Surgical intervention remains essential for patients with complicated obstruction, failed conservative therapy, or signs of strangulation or peritonitis. The conservative approach, which includes bowel rest, nasogastric decompression, intravenous fluid resuscitation and correction of electrolyte imbalances, has been shown to be effective in managing acute intestinal obstruction in many cases. Recent studies have reported success rates of conservative management ranging from 70% to 80%, with a reduction in the need for surgical intervention. Studies have reported that more than 70% of patients with adhesive small bowel obstruction were managed nonoperatively, with a low rate of delayed surgical intervention [6, 7]. However, conservative management is not without its limitations. Certain factors, such as the presence of bowel ischemia, perforation, or failure of conservative measures, necessitate surgical intervention. In our study, 13.5% of patients in the conservative group required subsequent surgical intervention due to failure of conservative management. Similarly, a study by Maienza *et al.* (2023) found that emergency surgery was needed in a majority of patients with bowel ischemia or peritonitis; however, most adhesive small bowel obstructions could be managed nonoperatively [8]. Advancements in diagnostic imaging, particularly computed tomography (CT), have significantly improved the ability to identify patients who may benefit from conservative management. CT imaging plays a crucial role in identifying the site and cause of obstruction, as well as assessing for complications such as ischemia or perforation. Previous studies have emphasized the importance of early and accurate diagnosis in determining the appropriate management strategy for small bowel obstruction [9, 10]. The decision between conservative and surgical management should be individualized, taking into account factors such as the patient's clinical presentation, underlying comorbidities and the presence of complications. A clinical severity score developed by Wassmer *et al.* (2023) identified several factors associated with the need for surgical resection. This score may aid clinicians in stratifying patients and determining the most appropriate management approach [11].

## Conclusion:

Conservative management effectively resolved most cases of acute intestinal obstruction, leading to shorter hospital stays with favorable outcomes. Surgical intervention remained necessary for complicated or non-responsive cases. An individualized approach based on clinical presentation and disease severity is essential to optimize patient recovery and minimize complications.
